# Longitudinal and nonlinear relations of dietary and Serum cholesterol in midlife with cognitive decline: results from EMCOA study

**DOI:** 10.1186/s13024-019-0353-1

**Published:** 2019-12-30

**Authors:** Yu An, Xiaona Zhang, Ying Wang, Yushan Wang, Wen Liu, Tao Wang, Zhongsheng Qin, Rong Xiao

**Affiliations:** 1 School of Public Health, Capital Medical University, No.10 Xitoutiao, You An Men Wai, Beijing, 100069 China; 2 Jincheng People’s Hospital, Jincheng, China

**Keywords:** Cholesterol, Cognitive decline, Nonlinear

## Abstract

**Background:**

Previous studies regarding the cholesterol-cognition relationship in midlife have generated conflicting results. We thus investigated whether dietary and blood cholesterol were associated with cognitive decline.

**Methods:**

Participants were drawn from a large cohort study entitled the Effects and Mechanism Investigation of Cholesterol and Oxysterol on Alzheimer’s disease (EMCOA) study. We included 2514 participants who completed a selection of comprehensive cognitive tests and were followed for an average of 2.3 years. Blood concentrations of total cholesterol (TC), high-density lipoprotein cholesterol (HDL-C), low-density lipoprotein cholesterol (LDL-C) and triglycerides (TG) were assessed and dietary intakes were investigated by food frequency questionnaire (FFQ) at baseline. Apolipoprotein E (APOE) was genotyped by Kompetitive Allele Specific PCR (KASP) sequencing. Non-high-density lipoprotein cholesterol (Non-HDL-C) and LDL-C/HDL-C ratio were calculated. The longitudinal effects of dietary and blood cholesterol on risk of global cognitive decline (decrease in Montreal Cognitive Assessment (MoCA) > 2 points) were examined using Cox proportional hazards models. The nonlinear associations with global and domain-specific cognitive decline was evaluated with mixed effect linear models.

**Results:**

In Cox proportional hazards models, neither cholesterol nor egg intake was associated with a higher risk of accelerated global cognitive decline. In contrast, the higher serum concentrations of TC, LDL-C, non-HDL-C and LDL-C/HDL-C ratio were positively associated with accelerated global cognitive decline regardless of being evaluated continuously or categorically while higher HDL-C was positively associated with accelerated global cognitive decline only when being evaluated categorically (all *P* < 0.05). In mixed effect linear models, quadratic and longitudinal relations of dietary cholesterol and egg intakes to global cognition, processing speed and executive function were observed. Moreover, there were inverted U-shaped relations of HDL-C, with processing speed and executive function but U-shaped relations of HDL-C and LDL-C/HDL-C ratio with verbal memory. Adverse linear associations of higher LDL-C and LDL-C/HDL-C ratio with multiple cognitive comes were also revealed. Additionally adjusting for APOE genotype did not modify cholesterol-cognition associations. Dietary and serum cholesterol had variable associations with global and domain-specific cognitive decline across educational groups.

**Conclusion:**

Differential associations between dietary/serum cholesterol and cognitive decline across different domains of function were observed in a particular population of middle-aged and elderly Chinese. Interventions to improve cognitive reserve regarding dietary instruction and lipid management should be tailored according to specific target.

**Trial registration:**

EMCOA, ChiCTR-OOC-17011882, Registered 5th, July 2017-Retrospectively registered, http://www.medresman.org/uc/project/projectedit.aspx?proj=2610

## Background

An extensive yet conflicting research has documented longitudinal associations between serum cholesterol and prospective cognitive decline [1]. Meanwhile, a sparse prior literature has identified no associations between cholesterol/egg intakes and incident dementia or Alzheimer’s disease (AD) [2]. However, a recent article published in JAMA has concluded that higher consumption of cholesterol and eggs was significantly associated with higher risk of incident cardiovascular disease (CVD) in a dose-response manner [3]. Since CVD are known to predict the risk of dementia [4], the role of dietary and serum cholesterol in cognitive function and AD is not as clear cut.

An updating meta-analysis of 17 studies indicated divergent cholesterol–cognition associations [5]. When measured in midlife, higher serum cholesterol levels were associated with an increased risk of late-life cognitive decline, AD and other dementia. However, this risk relationship has not been extended to late life. Prior studies of increased late-life serum cholesterol and subsequent risk of incident cognitive dysfunction report either null results or protective associations [6]. There is evidence that decreased cholesterol levels may be a manifestation of underlying dementia-related neuropathology [7]. Therefore, a non-linear pattern of both high and low serum cholesterol is related to increased risk of cognitive decline or AD have been noted. Wendell et al. have observed non-linear longitudinal [8] and cross-sectional [9] associations between serum cholesterol levels and cognitive function in Baltimore Longitudinal Study of Aging. Our group have also reported such similar sex-specific, non-linear, cross-sectional associations [10]. Despite that, there was a lack of cohort data and hence we do not know if non-linear patterns were replicated in longitudinal settings. With respect to dietary cholesterol, Vincent et al. [11] have indicated from meta-regression analyses that there is a positive, nonlinear relation between the changes in LDL-C and dietary cholesterol, suggesting a complex network of interrelationships between dietary cholesterol, serum cholesterol, which may obscure the role of dietary cholesterol in cognitive function. Similar to serum cholesterol levels, associations of dietary cholesterol and cognitive impairment, AD or dementia are mixed, albeit limited [2, 12], suggesting a need for nonlinear examination.

The present study thus aimed to augment the current understanding of both serum and dietary cholesterol-cognition non-linear associations in our longitudinal settings—the Effects and Mechanism investigation of Cholesterol and Oxysterol on Alzheimer’s disease (EMCOA) study [13]. While the study serves as an extension of our group’s prior cross-sectional examination, to our knowledge, no prior study has directly addressed both serum and dietary cholesterol nonlinearly. We aimed to examine potential quadratic relations of multiple serum cholesterol levels (TC, TG, HDL-C and LDL-C), cholesterol and egg intake to global and domain-specific cognitive decline, which may be more sensitive and helpful to elucidate the impacts of cholesterol on brain integrity and function.

## Materials and methods

### Participants

**Fig. 1:**
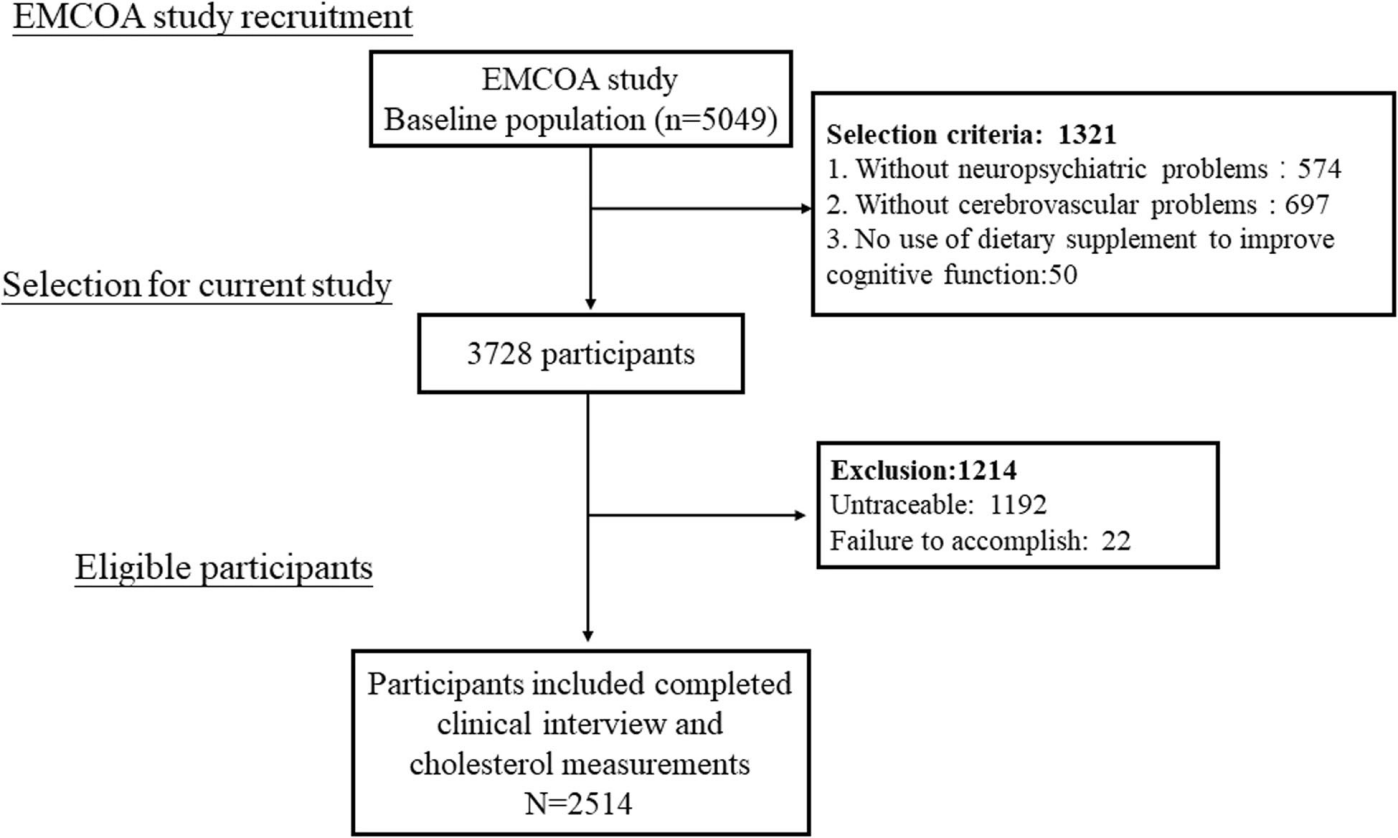
Study flow chart

Participants from the EMCOA study, a multicenter prospective study of community-dwelling volunteers initiated by Capital Medical University in 2014, returned to the respective research center in three locations approximately every 2 years. This study was registered at Chinese Clinical Trial Registry as ChiCTR-OOC-17011882. Beginning in 2014, participants between 50 to 70 years old were administered face-to-face interviews with the collection of sociodemographic information (e.g. age, sex and education years), medical history of chronic diseases, neuropsychological testing and dietary survey. Fasting venous blood samples were collected from the antecubital vein after a 12-h fast during all the interviews, following standardized protocols for storage of blood samples. The exclusion criteria for the original study included suffering from severe diseases or conditions known to affect cognitive function (e.g. depression, malignant tumors, a history of traumatic brain injury, cerebral infarction or cerebrovascular disease, long-term frequency intake of drugs and medication or dietary supplement to improve cognitive function). Finally, longitudinal data from 2514 middle-aged and elderly participants entered the study and were used for this analysis (Fig. 1). Because the EMCOA used continuous enrollment procedures, participants have different numbers of visits and follow times are also variable with a median time of 2.3 years. The medical Ethics Committee of Capital Medical University (No. 2013SY35) approved the study protocol and written informed consents were obtained from all subjects.

### Cognitive tests

At each study visit, standard cognitive tests were administered by trained study personnel in a standard order in a quiet room. The Montreal Cognitive Assessment (MoCA) [14] were used for global cognitive evaluation. Symbol Digit Modalities Test (SDMT) [15] was used to assess processing speed. The Auditory Verbal Learning Test (AVLT) [16] including 5 trials of recall of 12-word list measured immediate recall (AVLT-IR), short recall (AVLT-SR) and long recall (AVLT-LR) of memory. Logical Memory Test (LMT) [17] and Digit Span Forwards (DSF) [18] of Wechsler Memory Scale—Revised, Chinese version (WMS-RC) were used to measure attention and executive function respectively.

### Dietary assessment

Detailed dietary information at baseline was collected using food frequency questionnaire (FFQ) that asked about habitual intake of foods over the past year. Nutrients and energy intake were derived by multiplying the nutrients and energy content of each food of the specific portion size by the frequency of consumption as stated on the FFQ and then summed over all food items from the China Food Composition Database [12]. Consumption frequencies of food items were converted into estimated number per day using the middle value (eg 3–4 times per week = 0.5 times per day). Estimated daily total energy (in kJ/d), eggs (in g/d), cholesterol (in mg/d), carbohydrate, fat, saturated fatty acid (SFA), polyunsaturated fatty acid (PUFA), monounsaturated fatty acid (MUFA) and protein (in g/d) intakes were derived. All the values of dietary nutrients were energy adjusted according to the regression–residual method [19].

### Covariates

Sociodemographic information included age at study baseline, gender, education (in years). Lifestyle factors included smoking status (determined by self-report and dichotomized as current smoker or not). Risk factors for cognitive decline included body mass index (the ratio of weight to squared height, BMI), diabetes (fasting glucose≥7.0 mmol/L or antidiabetic medication), hypertension (measured blood pressure > 140/90 mmHg or antihypertensive medication), coronary heart disease (CHD) and per se use of a lipid-lowering medication (yes or no).

### Laboratory analysis

Centralized measurements of baseline fasting serum cholesterol levels were measured by enzymatic method (TC and TG) or direct method (HDL-C and LDL-C) using Automatic Biochemistry Analyzer (Olympus AU480, Japan) and commercially available diagnostic kits (Intec Products, Xiamen, China) in Beijing. Non-HDL-C was calculated by subtracting HDL-C from TC. The desirable concentrations of TC, TG and LDL-C are respectively less than 5.20 mmol/L, 1.70 mmol/L and 3.12 mmol/L. The reference range of HDL-C concentration and LDL-C/HDL-C ratio are respectively 1.04–1.7 mmol/L and 1.31–3.19. Less than 3.4 mmol/L of non-HDL-C concentration is considered normal [20].

### APOE genotyping

Identification and measurement of the APOE genotype with none ε4 (ε2/ε3, ε2/ε2, ε3/ε3), one ε4 (ε3/ε4, ε2/ε4) and two ε4 (ε4/ε4) in this study were achieved through the KASP genotyping assay by BioMiao Biological Technology, Beijing, China. In brief, leukocyte total genomic DNA was extracted from 400 μL of peripheral blood samples by using the Whole Blood DNA Extraction Kit (QIAamp® DNA Blood Mini Kit). DNA samples were then randomly placed on batches of 96-well plate. Genotyping of APOE was performed according to the manufacturer’s instructions [21]. For the purpose of quality control, 5% of samples were repeated and non-template controls were set in each plate.

### Statistical analysis

Statistical analyses were performed using STATA version 13.0 (STATA, College Station, TX). Prior to analysis, the normality of data distribution was checked. Continuous variables were expressed as medians (interquartile ranges, IQR) when non-normally distributed or the mean ± standard deviation (SD) when normally distributed. Analysis of variance (ANOVA) or the Kruskal-Wallis rank test was used for continuous variables as appropriate. The energy-adjusted values according to the residual method for all of the nutrients and eggs were calculated. The differences in frequencies of the categorical variables were evaluated using chi-square test or Fisher’s exact test. Dietary intakes of cholesterol and eggs were categorized into quartiles. The serum levels of cholesterol were categorized into two or three groups according to reference value or range. Cox proportional hazards regression models were used to estimate hazards ratios (HRs) for accelerated cognitive decline (a decrease in MoCA > 2 points between follow-up and baseline [22]) in baseline cholesterol and egg intakes as well as serum cholesterol. Longitudinal associations of dietary and serum cholesterol with global and domain-specific cognitive decline were estimated using linear mixed-effect models. We included quadratic terms in mixed-effect models when exploring the nonlinearity of the association between continuous baseline dietary cholesterol and egg intake as well as serum cholesterol concentrations and subsequent cognitive change. Each cognitive test was entered as a single outcome variable in separate mixed-effects regression models.

Covariates in the models were selected based on established and previously published risk factors for AD or associations with exposures in the current analysis. Model 1 included age, sex, years of education, BMI, smoking and drinking status, diabetes, hypertension and CHD history, per se use of lipid-lowering medication and intakes of energy, protein, carbohydrates, fat, cholesterol, SFA, PUFA and MUFA. Model 2 was adjusted as for model 1 and mutually for number of APOE ε4 alleles to test specially whether APOE genotype of the subjects have an influence on the identified relationship between cholesterol and various cognitive functions. Moreover, further subanalysis by four educational group, Elementary school (≤6 years of education), Junior middle school (7–9 years of education), Senior middle school (10–12 years of education) and College and above (at least college or university; ≥13 years of education) [13], were performed taking generally lower educational background into consideration. A two-sided *P* < 0.05 were considered statistically significant.

## Results

Of 2514 participants, 54.0% were women. The median age was 59 years and the median education years was 9 years. Median cholesterol and egg intakes were respectively 282.83 mg/d and 45.21 g/d. Compared with those with a lower cholesterol intake, participants with a higher cholesterol intake were less likely to be women, have hypertension and lipid-lowering medication per se use, have lower energy and carbohydrates intakes, but more educated, more likely to drink and have diabetes (all *P* < 0.05, Table 1). They also had higher protein, fat, SFA, PUFA, MUFA and egg intakes but lower serum TG concentrations (*P* < 0.05). In regard to baseline cognitive performance, participants with higher cholesterol intake performed significantly better than that with lower intake (all *P* < 0.05) except for AVLT-SR and AVLT-LR. No differences were observed with other covariates. Particularly, no significant differences emerged with respect to APOE ε4 allele, the distribution of which with higher ε2 and lower ε4 differed greatly from European and American populations [23].

**Table 1:** Baseline characteristics according to cholesterol intakes in 2514 participants in the EMCOA

	Cholesterol intake quartile, mg/d				*P* value
	Q1(< 188)	Q2 (188–283)	Q3 (283–385)	Q4 (> 385)	
Demographic characteristics					
Age	58 (56, 62)	59 (55, 62)	59 (55, 62)	59 (55, 62)	0.707
Women, n(%)	349 (55.57%)	362 (57.37%)	355 (56.71%)	291 (46.26%)	< 0.001*
Education years	9 (9, 12)	9 (9, 12)	12 (9, 12)	12 (9, 12)	0.001*
BMI (kg/m2)	24.6 (22.9, 26.7)	24.6 (22.6, 26.6)	24.4 (22.6, 26.4)	24.5 (22.6, 26.5)	0.371
Lifestyle					
Current smoker, n(%)	161 (25.64%)	151 (23.93%)	131 (20.93%)	154 (24.48%)	0.242
Current drinker, n(%)	143 (22.77%)	140 (22.19%)	151 (24.12%)	195 (31.00%)	0.001*
Medical History					
Diabetes, n(%)	69 (10.99%)	73 (11.57%)	96 (15.34%)	140 (22.26%)	< 0.001*
Hypertension, n(%)	235 (37.42%)	212 (33.60%)	195 (31.15%)	187 (29.73%)	0.022*
CHD, n(%)	79 (12.58%)	63 (9.98%)	52 (8.31%)	55 (8.74%)	0.051
vLipid-lowering medication per se use, n(%)	80 (12.74%)	74 (11.73%)	52 (8.31%)	57 (9.06%)	0.030*
APOE genotype with 0/1/2 ɛ4 risk alleles					0.473
0 (ε2/ε3, ε2/ε2, ε3/ε3)	525 (83.60%)	517 (81.93%)	515 (82.27%)	527 (83.78%)	
1 (ε3/ε4, ε2/ε4)	98 (15.61%)	109 (17.27%)	102 (16.29%)	100 (15.90%)	
2 (ε4/ε4)	5 (0.80%)	5 (0.79%)	9 (1.44%)	2 (0.32%)	
Dietary intakes†					
Energy, kJ/d	7165 (5732, 9169)	6477 (5005, 8524)	6927 (5691, 8135)	6823 (5159, 9034)	< 0.001*
Carbohydrates, g/d	271.88 (231.75, 310.47)	245.47 (214.74, 278.66)	244.60 (213.94, 274.34)	224.99 (197.91, 251.91)	< 0.001*
Protein, g/d	59.40 (52.85, 66.49)	63.22 (57.85, 69.18)	65.24 (59.68, 71.10)	71.69 (65.79, 80.71)	< 0.001*
Fat, g/d	59.29 (44.18, 74.88)	67.37 (54.51, 79.38)	66.69 (55.59, 79.09)	70.34 (59.43, 80.46)	< 0.001*
SFA, g/d	15.83 (12.50, 18.71)	18.63 (15.71, 21.42)	19.08 (16.56, 21.93)	21.87 (19.55, 24.87)	< 0.001*
PUFA, g/d	25.27 (16.77, 33.38)	27.88 (20.90, 34.62)	26.16 (19.34, 33.59)	23.69 (16.41, 31.20)	< 0.001*
MUFA, g/d	20.98 (15.27, 25.35)	24.16 (19.39, 28.39)	24.03 (20.06, 28.55)	27.68 (23.47, 32.52)	< 0.001*
Eggs, g/d	12.01 (4.01, 20.75)	29.97 (23.47, 41.12)	58.00 (49.11, 61.11)	62.66 (57.16, 66.61)	< 0.001*
Serum Cholesterol					
TC, mmol/L	4.51 (3.79, 5.19)	4.50 (3.80, 5.23)	4.51 (3.74, 5.25)	4.67 (3.98, 5.34)	0.054
TC > 5.20 mmol/L, n(%)	150 (23.92%)	160 (25.36%)	170 (27.16%)	177 (28.27%)	0.308
TG, mol/L	1.50 (1.09, 2.08)	1.59 (1.11, 2.13)	1.40 (1.06, 2.00)	1.36 (0.99, 2.00)	< 0.001*
TG > 1.70 mmol/L, n(%)	247 (39.39%)	277 (43.90%)	219 (34.98%)	224 (35.78%)	0.004*
HDL-C, mmol/L	1.24 (1.05, 1.48)	1.26 (1.10, 1.46)	1.30 (1.10, 1.50)	1.29 (1.10, 1.50)	0.06
HDL-C < 1.04 mmol/L, n(%)	146 (25.13%)	113 (19.25%)	111 (19.68%)	115 (20.35%)	0.052
HDL-C > 1.70 mmol/L, n(%)	46 (9.56%)	44 (8.49%)	62 (12.04%)	61 (11.94%)	0.166
LDL-C, mmol/L	2.77 (2.20, 3.31)	2.79 (2.20, 3.35)	2.72 (2.12, 3.31)	2.82 (2.30, 3.39)	0.438
LDL-C > 3.12 mmol/L, n(%)	206 (32.85%)	207 (32.81%)	209 (33.39%)	221 (35.30%)	0.762
Non-HDL, mmol/L	3.24 (2.62, 3.81)	3.20 (2.59, 3.92)	3.22 (2.58, 3.85)	3.35 (2.71, 3.93)	0.100
Non-HDL ≥ 3.40, n(%)	270 (43.06%)	262 (41.52%)	255 (40.73%)	296 (47.28%)	0.088
LDL-C/HDL-C	2.22 (1.68, 2.73)	2.20 (1.73, 2.71)	2.11 (1.72, 2.60)	2.18 (1.71, 2.71)	0.325
LDL-C/HDL-C ≤ 1.31, n(%)	50 (8.85%)	53 (9.20%)	63 (10.92%)	54 (9.52%)	0.654
LDL-C/HDL-C ≥ 3.19, n(%)	62 (10.75%)	55 (9.52%)	49 (8.70%)	59 (10.31%)	0.667
Baseline Cognitive Performance					
MoCA	24 (22, 26)	25 (22, 26)	25 (22, 27)	26 (24, 27)	< 0.001*
AVLT-IR	14 (11, 18)	14 (11, 18)	15 (12, 18)	15 (12, 19)	0.004*
AVLT-SR	5 (3, 7)	5 (3, 7)	5 (3, 7)	5 (4, 7)	0.154
AVLT-LR	4 (2, 6)	4 (2, 6)	4 (2, 6)	4 (3, 6)	0.087
SDMT	32 (25, 40)	34 (28, 41)	34 (26, 42)	35 (29, 44)	< 0.001*
DSF	8 (7, 8)	8 (7, 9)	8 (7, 9)	8 (7, 9)	< 0.001*
LMT	9.5 (5.5, 14.0)	10.0 (6.0, 14.0)	11.0 (6.5, 15.0)	11.0 (7.5, 15.0)	< 0.001*

Abbreviations: *MoCA* Montreal Cognitive Assessment, *AVLT-IR* auditory verbal learning test-immediate recall, *AVLT-SR* auditory verbal learning test-short recall, *AVLT-LR* auditory verbal learning test-long recall, *SDMT* symbol digit modalities test, *LMT* logical memory test, *DSF* digit span forwards, *DSB* digit span backwards, *BMI* body mass index, *CHD* coronary heart disease, *TC* total cholesterol, *HDL-C* high-density lipoprotein cholesterol, *LDL-C* low-density lipoprotein cholesterol, *TG* triglycerides, *Non-HDL-C* non-high-density lipoprotein cholesterol, *APOE* apolipoprotein E

Data shown as median (interquartile range) were compared between 4 groups using Kruskal-Wallis rank test;

Data shown as n (%) were compared between 4 groups using the chi-square test or Fisher’s exact test

†All dietary nutrients and egg intake are energy adjusted according to the regression–residual method

∗ *P* < 0.05

**Fig. 2:**
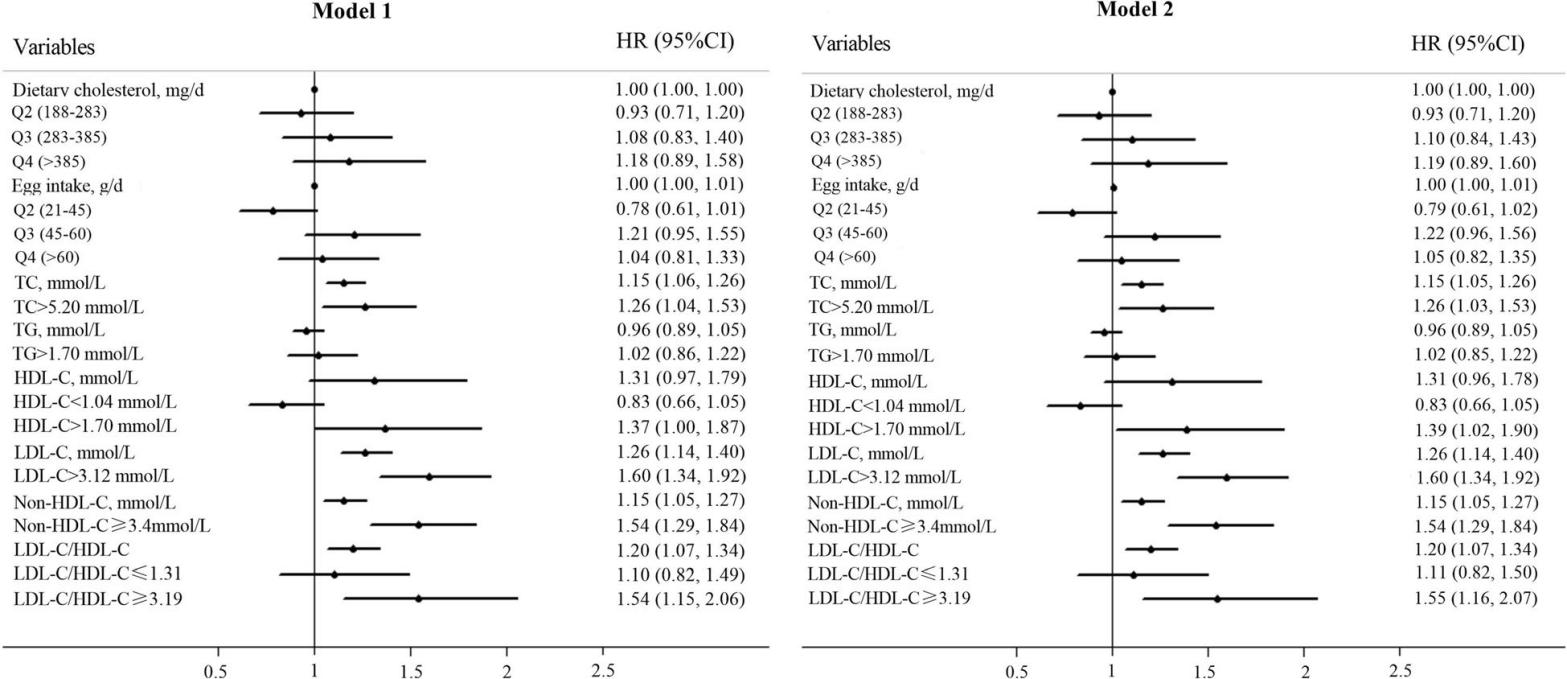
Forest plot for Cox proportional hazards model1 and model 2. HR: hazards ratio; CI: confidence interval. Model 1 was adjusted for sex, age, education years, BMI, smoking and drinking status, diabetes, hypertension and coronary artery disease history and per se use of lipid-lowering medication and intakes of energy, protein, carbohydrates, fat, SFA, PUFA, and MUFA. Model 2 was adjusted as for model 1 and for number of APOE ε4 alleles

During a median follow-up of 2.3 years, 546 participants (21.7%) were defined as accelerated cognitive decline. In multivariable Cox proportional hazards regression model 1 adjusted for AD risk factors (Table 2), neither cholesterol nor egg intake was associated with risk of accelerated cognitive decline regardless of being evaluated continuously (cholesterol: HR: 1.0002; 95% CI: 0.9995–1.0009; *P* = 0.590; egg: HR:1.002; 95% CI: 0.999–1.006; *P* = 0.128) or in quartiles (cholesterol: HR for highest compared with lowest quartiles: 1.18; 95% CI: 0.89–1.58; *P* = 0.256; egg: HR for highest compared with lowest quartiles: 1.04; 95% CI: 0.81–1.33; *P* = 0.786). With respect to serum cholesterol levels, additional serum concentrations of TC, LDL-C, non-HDL-C and LDL-C/HDL-C ratio were significantly associated with accelerated global cognitive decline when being evaluated continuously (HR for TC: 1.15, 95% CI: 1.06–1.26, *P* = 0.002; HR for LDL-C:1.26, 95% CI: 1.14–1.40, *P* < 0.001; HR for non-HDL-C: 1.15, 95%CI: 1.05–1.27, *P* = 0.004; HR for LDL-C/HDL-C ratio: 1.20, 95%CI: 1.07–1.34, P = 0.002). After being dichotomized, serum cholesterol was associated with an HR of 1.26 (95% CI: 1.04–1.53, *P* = 0.020) for TC higher than 5.20 mmol/L, 1.37 (95%CI: 1.00–1.87, *P* = 0.048) for HDL-C higher than 1.70 mmol/L, 1.60 (95% CI: 1.34–1.92, *P* < 0.001) for LDL-C higher than 3.12 mmol/L, 1.54 (95% CI: 1.29–1.84, *P* < 0.001) for non-HDL-C higher than 3.40 mmol/L and 1.54 (95% CI: 1.15–2.06, *P* = 0.003) for LDL-C/HDL-C ratio higher than 3.19 with significant adverse impact on global cognitive decline (Fig. 2). Their effect size changed only modestly without loss of significance if APOE genotype was included in the model. Therefore, the number of APOE ε4 risk alleles did not modify the association of either cholesterol intake or serum cholesterol levels with risk of global cognitive decline (Table 2, Fig. 2).

**Table 2:** Risk of accelerated cognitive decline in dietary and serum cholesterol levels in 2514 participants in the EMCOA

Variables	Model 1		Model 2	
	HR (95% CI)	*P* value	HR (95% CI)	*P* value
Dietary cholesterol, mg/d	1.0002 (0.9995–1.0009)	0.59	1.0002 (0.9995–1.0009)	0.564
Q1(< 188)	Ref		Ref	
Q2 (188–283)	0.93 (0.71–1.20)	0.558	0.93 (0.71–1.20)	0.569
Q3 (283–385)	1.08 (0.83–1.40)	0.571	1.10 (0.84–1.43)	0.488
Q4 (> 385)	1.18 (0.89–1.58)	0.256	1.19 (0.89–1.60)	0.231
Egg intake, g/d	1.002 (0.999–1.006)	0.128	1.003 (0.999–1.006)	0.111
Q1(< 21)	Ref		Ref	
Q2 (21–45)	0.78 (0.61–1.01)	0.058	0.79 (0.61–1.02)	0.068
Q3 (45–60)	1.21 (0.95–1.55)	0.125	1.22 (0.96–1.56)	0.108
Q4 (> 60)	1.04 (0.81–1.33)	0.786	1.05 (0.82–1.35)	0.703
Serum cholesterol, mmol/L				
TC, mmol/L	1.15 (1.06–1.26)	0.002*	1.15 (1.05–1.26)	0.002*
TC ≤ 5.20 mmol/L	Ref		Ref	
TC > 5.20 mmol/L	1.26 (1.04–1.53)	0.020*	1.26 (1.03–1.53)	0.022*
TG, mol/L	0.96 (0.89–1.05)	0.382	0.96 (0.89–1.05)	0.377
TG ≤ 1.70 mmol/L	Ref		Ref	
TG > 1.70 mmol/L	1.02 (0.86–1.22)	0.798	1.02 (0.85–1.22)	0.818
HDL-C, mmol/L	1.31 (0.97–1.79)	0.083	1.31 (0.96–1.78)	0.088
1.70 ≥ HDL-C ≥ 1.04 mmol/L	Ref		Ref	
HDL-C < 1.04 mmol/L	0.83 (0.66–1.05)	0.114	0.83 (0.66–1.05)	0.127
HDL-C > 1.70 mmol/L	1.37 (1.00–1.87)	0.048*	1.39 (1.02–1.90)	0.040*
LDL-C, mmol/L	1.26 (1.14–1.40)	< 0.001*	1.26 (1.14–1.40)	< 0.001*
LDL-C ≤ 3.12 mmol/L	Ref		Ref	
LDL-C > 3.12 mmol/L	1.60 (1.34–1.92)	< 0.001*	1.60 (1.34–1.92)	< 0.001*
Non-HDL-C, mmol/L	1.15 (1.05–1.27)	0.004*	1.15 (1.05–1.27)	0.004*
Non-HDL-C < 3.4 mmol/L	Ref		Ref	
Non-HDL-C ≥ 3.4 mmol/L	1.54 (1.29–1.84)	< 0.001*	1.54 (1.29–1.84)	< 0.001*
LDL-C/HDL-C	1.20 (1.07–1.34)	0.002*	1.20 (1.07–1.34)	0.002*
1.31 < LDL-C/HDL-C < 3.19	Ref		Ref	
LDL-C/HDL-C ≤ 1.31	1.10 (0.82–1.49)	0.521	1.11 (0.82–1.50)	0.506
LDL-C/HDL-C ≥ 3.19	1.54 (1.15–2.06)	0.003*	1.55 (1.16–2.07)	0.003*

Abbreviations: *TC* total cholesterol, *HDL-C* high-density lipoprotein cholesterol, *LDL-C* low-density lipoprotein cholesterol, *TG* triglycerides, *Non-HDL-C* non-high-density lipoprotein cholesterol, *HR* hazards ratio

Values were obtained from Cox proportional hazards regression models

Model 1 was adjusted for sex, age, education years, BMI, smoking and drinking status, diabetes, hypertension and coronary artery disease history and per se use of lipid-lowering medication and intakes of energy, protein, carbohydrates, fat, SFA, PUFA, and MUFA

Model 2 was adjusted as for model 1 and for number of APOE ε4 alleles

∗*P* < 0.05

**Fig. 3:**
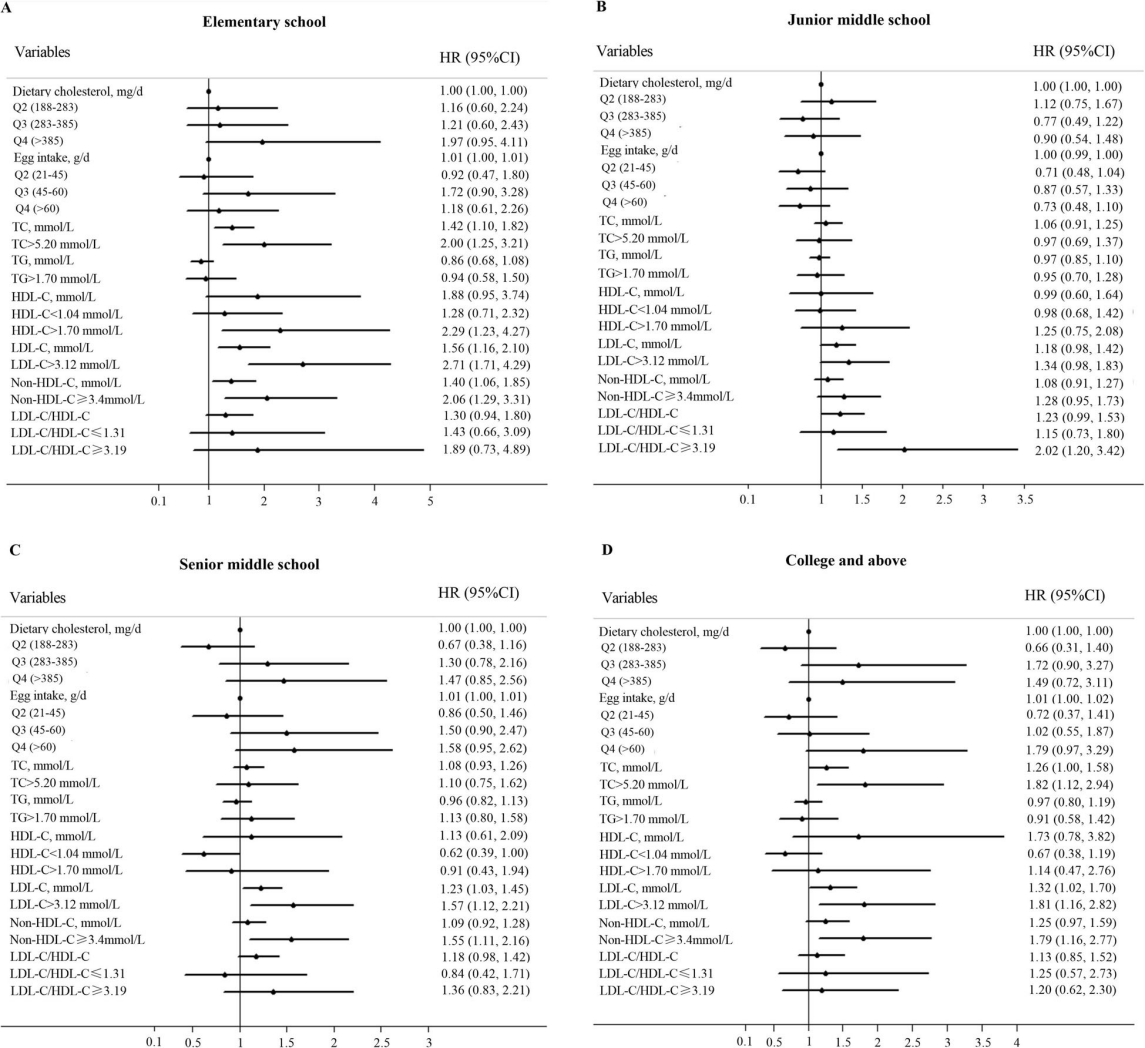
Forest plot of subanalysis for Elementary school (**a**), Junior middle school (**b**), Senior middle school (**c**), College and above (**d**) in Cox proportional hazards. HR: hazards ratio; CI: confidence interval

Table 3 demonstrated significant differences among different educational groups regarding to cholesterol and egg intakes (*P* = 0.0001), serum TG (*P* = 0.0145), HDL-C (*P* = 0.0413) and ratio of LDL-C/HDL-C (*P* = 0.0089). Generally, subjects with more years of education had more dietary cholesterol and egg intake and higher ratio of LDL-C/HDL-C. Subanalysis were thus performed and the whole population was stratified by different educational groups (Table 4, Fig. 3). Table 4 showed that higher serum cholesterol levels still increased risk of accelerated global cognitive decline generally but had some differences across different educational groups. Contrary to that, associations of dietary cholesterol and egg intake with cognitive decline in subanalysis differed from that in general analysis when being evaluated continuously. Dietary cholesterol had a trend toward lower risk of cognitive decline in Junior middle school group but higher risk in College and above group whereas egg intake was significantly associated with higher risk of cognitive decline in both Senior middle school group and College and above group.

**Table 3:** Comparison of dietary and serum cholesterol levels by different educational groups

Variables	Elementary school (*n* = 418)	Junior middle school (*n* = 925)	Senior middle school (*n* = 764)	College and above (*n* = 407)	*P* value
Dietary cholesterol, mg/d	255.5 (160.3, 367.0)	262.2 (176.5, 370.2)	296.0 (207.8, 392.7)	327.7 (212.8, 412.2)	0.0001*
Egg intake, g/d	39.6 (18.3, 59.5)	35.8 (18.3, 59.0)	48.4 (23.7, 60.4)	52.8 (25.8, 60.9)	0.0001*
TC, mmol/L	4.61 (3.90, 5.20)	4.51 (3.80, 5.28)	4.53 (3.78, 5.20)	4.62 (3.83, 5.30)	0.518
TG, mol/L	1.40 (1.00, 1.90)	1.48 (1.02, 2.05)	1.49 (1.10, 2.15)	1.45 (1.07, 2.10)	0.0145*
HDL-C, mmol/L	1.30 (1.10, 1.50)	1.27 (1.10, 1.50)	1.24 (1.09, 1.43)	1.26 (1.10, 1.47)	0.0413*
LDL-C, mmol/L	2.80 (2.24, 3.30)	2.76 (2.13, 3.30)	2.76 (2.20, 3.39)	2.86 (2.27, 3.44)	0.0962
Non-HDL-C, mmol/L	3.29 (2.70, 3.83)	3.22 (2.61, 3.85)	3.25 (2.56, 3.88)	3.26 (2.72, 3.95)	0.5925
LDL-C/HDL-C	2.13 (1.72, 2.57)	2.15 (1.67, 2.67)	2.22 (1.73, 2.75)	2.25 (1.75, 2.77)	0.0089*

Abbreviations: *TC* total cholesterol, *HDL-C* high-density lipoprotein cholesterol, *LDL-C* low-density lipoprotein cholesterol, *TG* triglycerides, *Non-HDL-C* non-high-density lipoprotein cholesterol

Elementary school: ≤6 years of education; Junior middle school: 7–9 years of education; Senior middle school: 10–12 years of education; College and above: at least college or university; ≥13 years of education)

∗*P* < 0.05

**Table 4:** Subanalysis for risk of accelerated cognitive decline in dietary and serum cholesterol levels by different educational groups

Variables	Elementary school (*n* = 418)		Junior middle school (*n* = 925)		Senior middle school (*n* = 764)		College and above (*n* = 407)	
	HR (95% CI)	*P* value	HR (95% CI)	*P* value	HR (95% CI)	*P* value	HR (95% CI)	*P* value
Dietary cholesterol, mg/d	1.002 (0.9999–1.0034)	0.063	0.999 (0.998–0.99998)	0.046*	1.001 (0.999–1.003)	0.077	1.002 (1.000–1.003)	0.041*
Q1(< 188)	Ref		Ref		Ref		Ref	
Q2 (188–283)	1.16 (0.60–2.24)	0.666	1.12 (0.75–1.67)	0.57	0.67 (0.38–1.16)	0.153	0.66 (0.31–1.40)	0.276
Q3 (283–385)	1.21 (0.60–2.43)	0.596	0.77 (0.49–1.22)	0.269	1.30 (0.78–2.16)	0.309	1.72 (0.90–3.27)	0.098
Q4 (> 385)	1.97 (0.95–4.11)	0.07	0.90 (0.54–1.48)	0.667	1.47 (0.85–2.56)	0.171	1.49 (0.72–3.11)	0.287
Egg intake, g/d	1.006 (0.998–1.014)	0.119	0.996 (0.991–1.001)	0.155	1.008 (1.002–1.014)	0.013*	1.010 (1.001–1.018)	0.020*
Q1(< 21)	Ref		Ref		Ref		Ref	
Q2 (21–45)	0.92 (0.47–1.80)	0.817	0.71 (0.48–1.04)	0.076	0.86 (0.50–1.46)	0.572	0.72 (0.37–1.41)	0.337
Q3 (45–60)	1.72 (0.90–3.28)	0.103	0.87 (0.57–1.33)	0.527	1.50 (0.90–2.47)	0.116	1.02 (0.55–1.87)	0.96
Q4 (> 60)	1.18 (0.61–2.26)	0.623	0.73 (0.48–1.10)	0.128	1.58 (0.95–2.62)	0.08	1.79 (0.97–3.29)	0.062
Serum cholesterol, mmol/L								
TC, mmol/L	1.42 (1.10–1.82)	0.006*	1.06 (0.91–1.25)	0.433	1.08 (0.93–1.26)	0.313	1.258 (1.002–1.578)	0.048*
TC ≤ 5.20 mmol/L	Ref		Ref		Ref		Ref	
TC > 5.20 mmol/L	2.00 (1.25–3.21)	0.004*	0.97 (0.69–1.37)	0.868	1.10 (0.75–1.62)	0.638	1.82 (1.12–2.94)	0.015*
TG, mol/L	0.86 (0.68–1.08)	0.183	0.97 (0.85–1.10)	0.622	0.96 (0.82–1.13)	0.643	0.97 (0.80–1.19)	0.783
TG ≤ 1.70 mmol/L	Ref		Ref		Ref		Ref	
TG > 1.70 mmol/L	0.94 (0.58–1.50)	0.786	0.95 (0.70–1.28)	0.742	1.13 (0.80–1.58)	0.491	0.91 (0.58–1.42)	0.669
HDL-C, mmol/L	1.88 (0.95–3.74)	0.071	0.99 (0.60–1.64)	0.977	1.13 (0.61–2.09)	0.705	1.73 (0.78–3.82)	0.177
1.70 ≥ HDL-C ≥ 1.04 mmol/L	Ref		Ref		Ref		Ref	
HDL-C < 1.04 mmol/L	1.28 (0.71–2.32)	0.414	0.98 (0.68–1.42)	0.922	0.62 (0.39–1.002)	0.051	0.67 (0.38–1.19)	0.169
HDL-C > 1.70 mmol/L	2.29 (1.23–4.27)	0.009*	1.25 (0.75–2.08)	0.399	0.91 (0.43–1.94)	0.806	1.14 (0.47–2.76)	0.763
LDL-C, mmol/L	1.56 (1.16–2.10)	0.003*	1.18 (0.98–1.42)	0.076	1.23 (1.03–1.45)	0.020*	1.32 (1.02–1.70)	0.032*
LDL-C ≤ 3.12 mmol/L	Ref		Ref		Ref		Ref	
LDL-C > 3.12 mmol/L	2.71 (1.71–4.29)	< 0.001*	1.34 (0.98–1.83)	0.07	1.57 (1.12–2.21)	0.009*	1.81 (1.16–2.82)	0.008*
Non-HDL-C, mmol/L	1.40 (1.06–1.85)	0.019*	1.08 (0.91–1.27)	0.394	1.09 (0.92–1.28)	0.325	1.25 (0.97–1.59)	0.081
Non-HDL-C < 3.4 mmol/L	Ref		Ref		Ref		Ref	
Non-HDL-C ≥ 3.4 mmol/L	2.06 (1.29–3.31)	0.003*	1.28 (0.95–1.73)	0.098	1.55 (1.11–2.16)	0.010*	1.79 (1.16–2.77)	0.009*
LDL-C/HDL-C	1.30 (0.94–1.80)	0.108	1.23 (0.99–1.53)	0.065	1.18 (0.98–1.42)	0.077	1.13 (0.85–1.52)	0.405
1.31 < LDL-C/HDL-C < 3.19	Ref		Ref		Ref		Ref	
LDL-C/HDL-C ≤ 1.31	1.43 (0.66–3.09)	0.363	1.15 (0.73–1.80)	0.544	0.84 (0.42–1.71)	0.636	1.25 (0.57–2.73)	0.583
LDL-C/HDL-C ≥ 3.19	1.89 (0.73–4.89)	0.19	2.02 (1.20–3.42)	0.008*	1.36 (0.83–2.21)	0.219	1.20 (0.62–2.30)	0.589

Abbreviations: *TC* total cholesterol, *HDL-C* high-density lipoprotein cholesterol, *LDL-C* low-density lipoprotein cholesterol, *TG* triglycerides, *Non-HDL-C* non-high-density lipoprotein cholesterol, *HR* hazards ratio

Values were obtained from Cox proportional hazards regression models adjusted for sex, age, BMI, smoking and drinking status, diabetes, hypertension and coronary artery disease history and per se use of lipid-lowering medication and intakes of energy, protein, carbohydrates, fat, SFA, PUFA, MUFA and number of APOE ε4 alleles

Elementary school: ≤6 years of education; Junior middle school: 7–9 years of education; Senior middle school: 10–12 years of education; College and above: at least college or university; ≥13 years of education**)**

∗*P* < 0.05

**Fig. 4:**
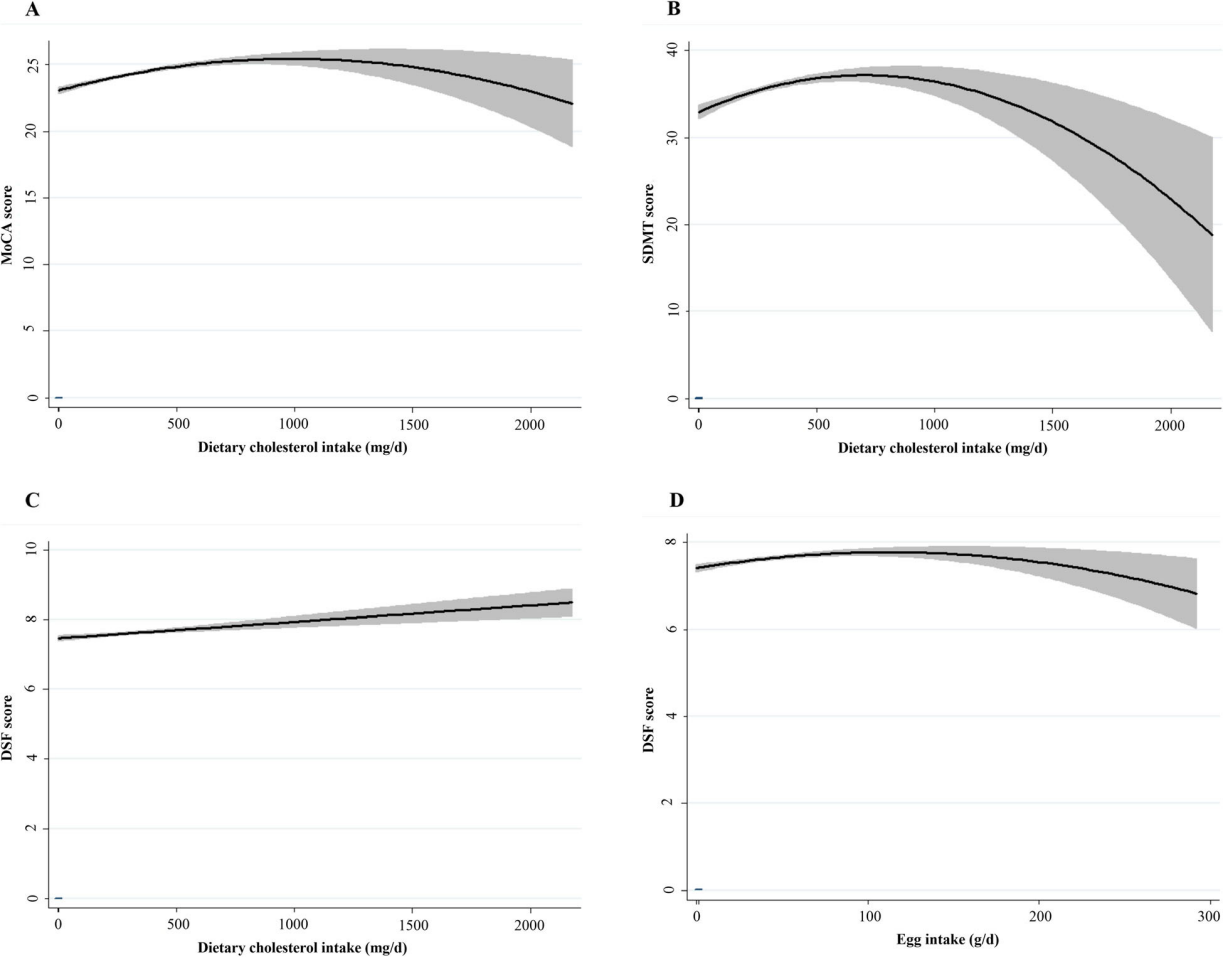
Significant effects of dietary cholesterol for MoCA (**a**), SDMT (**b**), DSF (**c**) and egg intake for DSF(**d**) in mixed-effect linear model 2. MoCA: Montreal Cognitive Assessment; SDMT: symbol digit modalities test; DSF: digit span forwards. Model 2 was adjusted as for sex, age, education years, BMI, smoking and drinking status, diabetes, hypertension and coronary artery disease history and per se use of lipid-lowering medication and intakes of energy, protein, carbohydrates, fat, SFA, PUFA, MUFA and number of APOE ε4 alleles

**Fig. 5:**
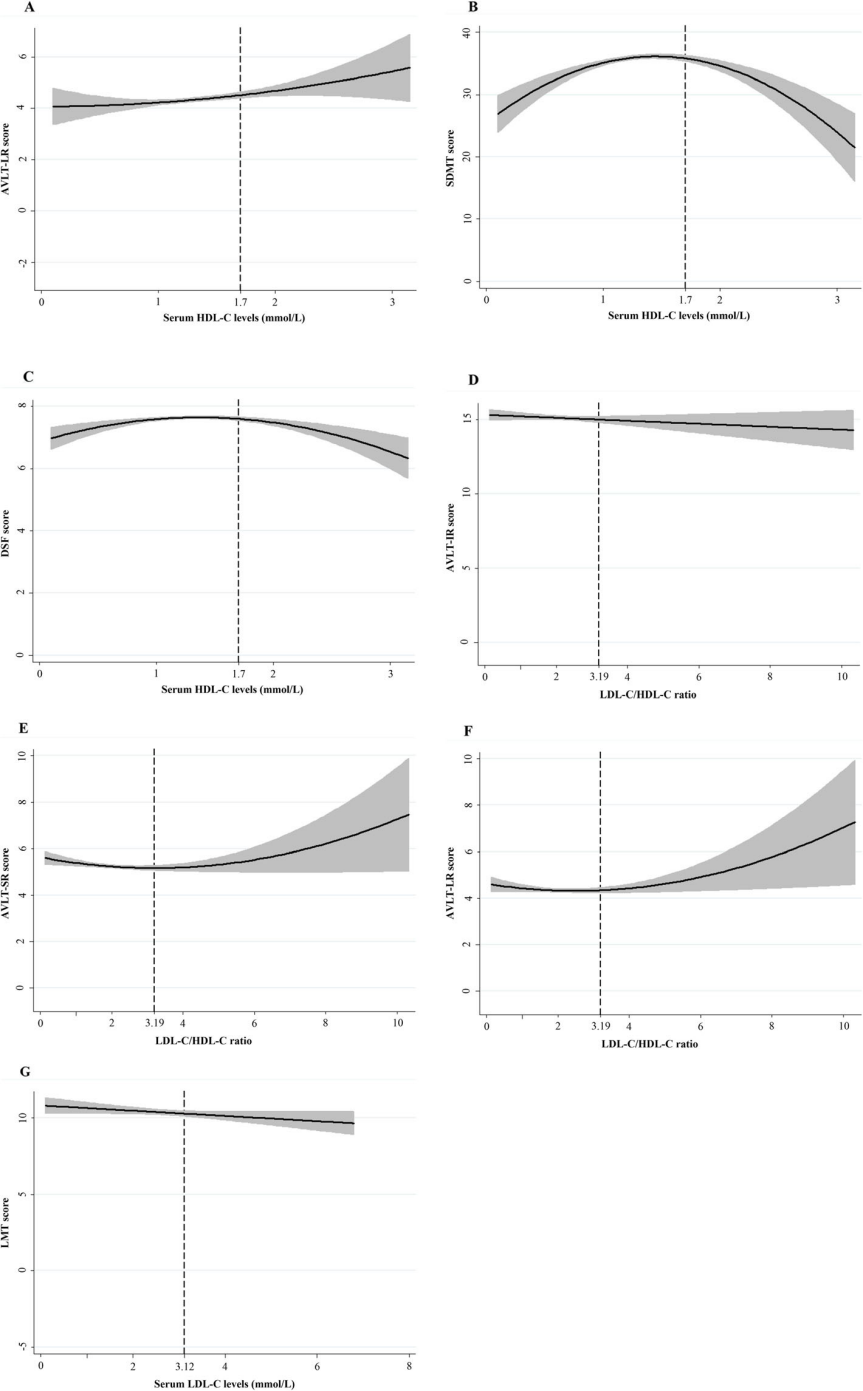
Significant effects of HDL-C for AVLT-LR (**a**), SDMT (**b**) and DSF (**c**), LDL-C/HDL-C ratio for AVLT-IR (**d**), AVLT-SR (**e**) and AVLT-LR (**f**) and LDL-C for LMT (**g**) in mixed-effect linear model 2. AVLT-IR: auditory verbal learning test-immediate recall; AVLT-SR: auditory verbal learning test-immediate recall; AVLT-LR: auditory verbal learning test-long recall; LMT: Logical Memory Test; SDMT: symbol digit modalities test; DSF: digit span forwards. TC: total cholesterol; HDL-C: high-density lipoprotein cholesterol; LDL-C: low-density lipoprotein cholesterol. Model 2 was adjusted as for sex, age, education years, BMI, smoking and drinking status, diabetes, hypertension and coronary artery disease history and per se use of lipid-lowering medication and intakes of energy, protein, carbohydrates, fat, SFA, PUFA, MUFA and number of APOE ε4 alleles .

Findings from mixed-effects linear regression analyses for dietary cholesterol and egg intake were shown in Table 5. Regarding nonlinear effects, significant longitudinal, quadratic effects of dietary cholesterol were identified for MoCA (β = − 0.00000142, *P* = 0.023, Fig. 4a) and SDMT (β = − 0.00000713, *P* = 0.001, Fig. 4b) and egg intake for DSF (β = − 0.000022, *P* = 0.008, Fig. 4d). Besides, positive linear associations of dietary cholesterol were identified for DSF (β = 0.005, *P* = 0.048, Fig. 4c). Table 6 demonstrated nonlinear or linear longitudinal associations of serum cholesterol with cognitive outcomes. No quadratic associations of TC, TG and Non-HDL-C with cognitive decline were observed. When it comes to HDL-C, mixed-effect linear models revealed significant U-shaped effects of HDL-C on AVLT-LR (β = 0.514, *P* = 0.045, Fig. 5a). Similarly, U-shaped patterns of ratio of LDL-C/HDL-C were also identified for AVLT-SR (β = 0.054, *P* = 0.032, Fig. 5e) and AVLT-LR (β = 0.054, *P* = 0.032, Fig. 5f). Besides, inverted U-shaped effects of HDL-C for SDMT (β = − 3.046, *P* = 0.004, Fig. 5b) and DSF (β = − 0.342, *P* = 0.006, Fig. 5c) were also identified, such that participants performed better at midrange HDL-C than at high and low levels. In Sion, the models also revealed adverse linear longitudinal effects of LDL-C for LMT (β = − 1.099, *P* = 0.028, Fig. 5g) and ratio of LDL-C/HDL-C for AVLT-IR (β = − 0.547, *P* = 0.047, Fig. 5d). Further adjustment of number of APOE ε4 risk alleles did not modify these associations. No significant effects arose for the remainder of serum cholesterol and cognitive tests.

**Table 5:** Results of mixed-effects regression models predicting cognitive test performance from dietary cholesterol and egg intake

Cognitive tests	Dietary cholesterol2		Dietary cholesterol		Egg intake2		Egg intake	
	β	*P* value	β	*P* value	β	*P* value	β	*P* value
Model 1								
MoCA	−0.00000142	0.023*	0.0020	0.001*	−0.0000197	0.331	0.0046	0.135
AVLT-IR	− 0.00000124	0.19	0.0018	0.052	−0.0000157	0.605	0.0030	0.508
AVLT-SR	−0.000000754	0.122	0.0006	0.223	−0.0000111	0.478	0.0003	0.895
AVLT-LR	−0.000000834	0.121	0.0006	0.28	−0.00000582	0.737	−0.0005	0.836
SDMT	− 0.00000713	0.001*	0.0066	0.003*	−0.0001176	0.097	0.0145	0.172
DSF	− 0.00000047	0.072	0.0005	0.048*	−0.000022	0.008*	0.0044	0.001*
LMT	−0.00000158	0.201	0.0023	0.064	−0.0000214	0.588	0.0073	0.225
Model 2								
MoCA	−0.00000142	0.023*	0.0020	0.001*	−0.0000198	0.328	0.0046	0.133
AVLT-IR	− 0.00000124	0.19	0.0018	0.053	−0.000016	0.598	0.0031	0.504
AVLT-SR	−0.000000751	0.122	0.0006	0.231	− 0.0000113	0.469	0.0003	0.893
AVLT-LR	−0.000000831	0.122	0.0006	0.288	−0.00000602	0.728	−0.0005	0.836
SDMT	−0.0000071	0.001*	0.0066	0.003*	−0.0001177	0.096	0.0145	0.174
DSF	−0.000000475	0.069	0.0005	0.045*	−0.0000221	0.008*	0.0044	< 0.001*
LMT	−0.00000157	0.205	0.0023	0.067	−0.0000215	0.587	0.0072	0.229

Abbreviations: *MoCA* Montreal Cognitive Assessment, *AVLT-IR* auditory verbal learning test-immediate recall, *AVLT-SR* auditory verbal learning test-short recall, *AVLT-LR* auditory verbal learning test-long recall, *SDMT* symbol digit modalities test, *DSF* digit span forwards, *LMT* logical memory test

β: unstandardized regression coefficients were obtained from mixed-effects regression models

Model 1 was adjusted for sex, age, education years, BMI, smoking and drinking status, diabetes, hypertension and coronary artery disease history and per se use of lipid-lowering medication and intakes of energy, protein, carbohydrates, fat, SFA, PUFA, and MUFA

Model 2 was adjusted as for model 1 and for number of APOE ε4 alleles

∗*P* < 0.05

**Table 6:** Results of mixed-effects regression models predicting cognitive test performance from serum cholesterol

Cognitive tests	TC^2^	TC	TG^2^	TG	HDL-C^2^	HDL-C	LDL-C^2^	LDL-C	Non-HDL-C^2^	Non-HDL-C	LDL-C/HDL-C^2^	LDL-C/HDL-C
	β	β	β	β	β	β	β	β	β	β	β	β
Model 1												
MoCA	0.024	−0.210	− 0.002	− 0.020	0.208	− 0.234	0.020	− 0.179	0.007	− 0.067	0.021	− 0.269
AVLT-IR	0.016	−0.157	0.000	−0.012	0.353	−0.677	0.094	−0.748	0.018	−0.159	0.050	−0.547*
AVLT-SR	0.014	−0.163	0.005	−0.034	0.270	−0.619	0.038	−0.337	0.018	−0.169	0.054*	−0.415*
AVLT-LR	0.021	−0.213	0.002	0.007	0.514*	−1.362	0.038	−0.337	0.025	−0.200	0.06*	−0.401*
SDMT	− 0.096	0.655	0.008	−0.130	−3.046*	8.662*	−0.248	1.181	−0.161	0.797	−0.147	0.311
DSF	−0.009	0.091	−0.002	0.010	− 0.342*	1.008*	−0.015	0.096	−0.023	0.167	−0.019	0.084
LMT	0.071	−0.848	−0.011	0.171	0.661	−2.015	0.133	−1.099*	0.053	−0.544	0.023	−0.457
Model 2												
MoCA	0.024	−0.216	−0.003	− 0.018	0.209	− 0.240	0.020	− 0.183	0.008	− 0.070	0.021	− 0.269
AVLT-IR	0.018	−0.178	0.000	−0.009	0.358	−0.699	0.097	− 0.767	0.020	−0.169	0.051	−0.553*
AVLT-SR	0.016	−0.183	0.005	−0.032	0.275	−0.640	0.041	−0.356	0.019	−0.180	0.055*	−0.423*
AVLT-LR	0.022	−0.230	0.002	0.009	0.518*	−1.380	0.048	−0.375	0.026	− 0.209	0.061*	− 0.409*
vSDMT	−0.094	0.620	0.008	−0.130	−3.036*	8.627*	−0.243	1.141	−0.160	0.780	−0.143	0.282
DSF	−0.009	0.092	−0.002	0.011	−0.343*	1.008*	−0.015	0.098	−0.023	0.167	− 0.020	0.088
LMT	0.073	−0.868	−0.011	0.170	0.667	−2.036	0.136	−1.123*	0.054	−0.554	0.026	−0.474

Abbreviations: *MoCA* Montreal Cognitive Assessment, *AVLT-IR* auditory verbal learning test-immediate recall, *AVLT-SR* auditory verbal learning test-short recall, *AVLT-LR* auditory verbal learning test-long recall, *SDMT* symbol digit modalities test, *DSF* digit span forwards, *LMT* logical memory test, *TC* total cholesterol, *HDL-C* high-density lipoprotein cholesterol, *LDL-C* low-density lipoprotein cholesterol, *TG* triglycerides, *Non-HDL-C* non- high-density lipoprotein cholesterol

β: unstandardized regression coefficients were obtained from mixed-effects regression models

Model 1 was adjusted for sex, age, education years, BMI, smoking and drinking status, diabetes, hypertension and coronary artery disease history and per se use of lipid-lowering medication and intakes of energy, protein, carbohydrates, fat, SFA, PUFA, and MUFA

Model 2 was adjusted as for model 1 and for number of APOE ε4 alleles

∗*P* < 0.05

Subgroup analysis by educational groups differed greatly from general analysis when it comes to nonlinear effects (Tables 7 and 8). U-shaped effects of serum multiple cholesterol measurements (TC, TG, HDL-C, LDL-C and Non-HDL-C) for all the cognitive performance except DSF were identified in Junior middle school group whereas only U-shaped effects of HDL-C and ratio of LDL-C/HDL-C for AVLT-SR and AVLT-LR remain significant in Senior middle school group. Moreover, only inverted U-shaped effects of HDL-C, LDL-C and ratio of LDL-C/HDL-C for MoCA, AVLT-SR and SDMT were observed in College and above group. No significant associations were demonstrated in Elementary school group.

**Table 7:** Subanalysis for results of mixed-effects regression models predicting cognitive test performance from dietary cholesterol and egg intake by different educational groups

Cognitive tests	Dietary cholesterol2		Dietary cholesterol		Egg intake2		Egg intake	
	β	*P* value	β	*P* value	β	*P* value	β	*P* value
Elementary school (*n* = 418)								
MoCA	0.00000347	0.257	−0.004239	0.102	0.0000902	0.202	−0.020563	0.048*
AVLT-IR	−0.00000105	0.746	−0.001233	0.654	0.0000432	0.56	−0.017803	0.107
AVLT-SR	0.00000115	0.501	−0.002667	0.067	0.0000348	0.375	−0.013017	0.026*
AVLT-LR	0.000000766	0.693	−0.002141	0.193	0.0000178	0.689	−0.011021	0.095
SDMT	0.00000511	0.519	−0.001092	0.87	0.0001744	0.342	−0.009431	0.726
DSF	0.000000705	0.537	−0.000568	0.557	0.00000138	0.958	−0.001005	0.796
LMT	0.00000044	0.933	−0.002083	0.641	0.0000577	0.624	−0.017358	0.328
Junior middle school (*n* = 925)								
MoCA	−0.00000223	0.064	0.002945	0.011*	−0.0000345	0.167	0.0077031	0.087
AVLT-IR	−0.00000349	0.062	0.004374	0.015*	−0.0000191	0.622	0.0079525	0.255
AVLT-SR	−0.00000199	0.038*	0.001977	0.032*	−0.0000191	0.336	0.0032397	0.366
AVLT-LR	−0.00000158	0.136	0.001501	0.138	−0.00000175	0.936	0.0001187	0.976
SDMT	−0.0000127	0.002*	0.010674	0.005*	−0.0001801	0.030*	0.0240724	0.108
DSF	−0.000000446	0.378	0.000669	0.167	−0.000023	0.028*	0.0051033	0.007*
LMT	−0.00000132	0.553	0.003593	0.091	−0.0000423	0.357	0.013091	0.114
Senior middle school (*n* = 764)								
MoCA	−0.00000155	0.075	0.002437	0.013*	−0.00000089	0.988	0.0045697	0.483
AVLT-IR	−0.00000141	0.336	0.002893	0.078	0.0000611	0.521	−0.001338	0.902
AVLT-SR	−0.00000127	0.091	0.000801	0.342	−0.00000153	0.975	− 0.001089	0.845
AVLT-LR	−0.00000132	0.112	0.000674	0.47	−0.0000122	0.822	0.0005769	0.926
SDMT	−0.0000018	0.606	0.002899	0.459	0.00000761	0.973	0.0012421	0.962
DSF	−0.000000936	0.011*	0.001025	0.013*	− 0.0000315	0.184	0.0068822	0.011*
LMT	−0.00000268	0.183	0.002725	0.226	0.0000091	0.944	0.0061439	0.679
College and above (*n* = 407)								
MoCA	−0.0000011	0.511	0.001583	0.309	−0.0001057	0.052	0.0134549	0.053
AVLT-IR	−0.00000213	0.544	0.001039	0.75	− 0.0002722	0.016*	0.0243167	0.092
AVLT-SR	0.000000363	0.839	0.000177	0.915	−0.0000809	0.16	0.0097477	0.186
AVLT-LR	0.000000472	0.811	0.000178	0.923	−0.0000889	0.162	0.0101086	0.214
SDMT	0.00000371	0.662	−0.005496	0.486	− 0.0000766	0.778	− 0.012119	0.728
DSF	−0.000000251	0.782	−0.000251	0.766	−0.0000345	0.241	0.0033226	0.378
LMT	0.00000291	0.47	−0.001744	0.639	−0.0001277	0.332	0.0196316	0.242

Abbreviations: *MoCA* Montreal Cognitive Assessment, *AVLT-IR* auditory verbal learning test-immediate recall, *AVLT-SR* auditory verbal learning test-short recall, *AVLT-LR* auditory verbal learning test-long recall, *SDMT* symbol digit modalities test, *DSF* digit span forwards, *LMT* logical memory test

β: unstandardized regression coefficients were obtained from mixed-effects regression models adjusted for sex, age, BMI, smoking and drinking status, diabetes, hypertension and coronary artery disease history and per se use of lipid-lowering medication, number of APOE ε4 alleles and intakes of energy, protein, carbohydrates, fat, SFA, PUFA, and MUFA

Elementary school: ≤6 years of education; Junior middle school: 7–9 years of education; Senior middle school: 10–12 years of education; College and above: at least college or university; ≥13 years of education)

∗*P* < 0.05

**Table 8:** Subanalysis for results of mixed-effects regression models predicting cognitive test performance from serum cholesterol by different educational groups

Cognitive tests	TC^2^	TC	TG^2^	TG	HDL-C^2^	HDL-C	LDL-C^2^	LDL-C	Non-HDL-C^2^	Non-HDL-C	LDL-C/HDL-C^2^	LDL-C/HDL-C
	β	β	β	β	β	β	β	β	β	β	β	β
Elementary school (*n* = 418)												
MoCA	− 0.128	1.102	− 0.072	0.366	1.411	−3.634	− 0.208	1.016	− 0.191	1.103	− 0.099	0.232
AVLT-IR	−0.027	0.205	0.037	− 0.287	0.071	0.002	−0.029	− 0.025	− 0.079	0.445	− 0.010	− 0.225
AVLT-SR	− 0.055	0.503	0.011	− 0.036	− 0.525	1.604	− 0.053	0.266	− 0.100	0.646	− 0.030	0.042
AVLT-LR	−0.061	0.599	− 0.018	0.115	−0.129	0.462	−0.102	0.630	−0.124	0.861	−0.044	0.208
SDMT	0.093	−1.779	− 0.210	0.802	−1.413	4.817	0.072	−1.138	0.026	−1.357	0.038	−1.229
DSF	−0.030	0.254	−0.010	0.106	−0.127	0.271	−0.088	0.468	−0.065	0.419	−0.106	0.513
LMT	0.259	−2.713	−0.110	1.212	2.315	−7.679	0.265	−2.009	0.052	−0.485	0.043	−0.343
Junior middle school (*n* = 925)												
MoCA	0.122*	−1.150*	−0.013	0.082	−0.108	0.598	0.149	−0.876	0.124*	−0.869*	0.021	−0.241
AVLT-IR	0.164*	−1.536*	−0.010	0.149	0.936	−2.163	0.225	−1.515*	0.186*	−1.306*	0.055	−0.646
AVLT-SR	0.083*	−0.824*	−0.001	0.058	0.762*	−2.188*	0.143*	−0.948*	0.101*	−0.730*	0.155	−0.832*
AVLT-LR	0.067	−0.669	−0.008	0.164	0.935*	−2.849*	0.149*	−0.958*	0.095*	−0.673*	0.177	−0.846
SDMT	−0.011	−0.501	0.058*	−0.833*	−2.383	6.464	−0.167	0.455	−0.009	− 0.586	−0.418	1.403
DSF	0.010	−0.068	−0.004	0.005	−0.354	1.059	0.047	−0.223	−0.015	0.126	−0.014	0.081
LMT	0.104	−1.216	−0.017	0.148	0.927	−3.183	0.297*	−1.925*	0.108	−0.940	−0.040	0.021
Senior middle school (*n* = 764)												
MoCA	0.008	0.019	0.006	−0.037	0.080	−0.183	0.029	−0.194	− 0.036	0.364	0.026	−0.153
AVLT-IR	0.022	−0.195	0.004	0.040	1.178	−3.704	0.139	−1.063	−0.010	0.121	0.059	−0.432
AVLT-SR	0.037	−0.389	0.011	−0.109	1.560*	−4.367*	0.038	−0.366	0.021	−0.184	0.062*	−0.436*
AVLT-LR	0.061	−0.585	0.008	−0.001	1.712*	−4.820*	0.062	−0.494	0.052	−0.348	0.069*	−0.441
SDMT	−0.133	1.337	−0.051	0.909	−1.483	3.480	−0.086	0.292	−0.303	2.237	−0.094	0.391
DSF	0.001	−0.010	−0.001	− 0.005	−0.192	0.654	−0.008	0.000	−0.018	0.121	−0.002	− 0.071
LMT	0.076	−0.858	−0.005	0.159	0.926	−2.362	0.142	−1.264	0.067	−0.624	0.095	−0.944
College and above (*n* = 407)												
MoCA	−0.026	0.237	0.025	−0.297	−0.796	2.968	−0.039	0.093	−0.031	0.140	−0.227*	0.747
AVLT-IR	−0.173	1.613	−0.010	−0.145	−2.518	7.657	−0.168	0.901	−0.181	1.140	−0.325	1.223
AVLT-SR	−0.084	0.790	−0.006	−0.047	−1.559*	4.756*	−0.080	0.414	−0.080	0.499	−0.165	0.581
AVLT-LR	−0.061	0.500	0.005	−0.196	−0.524	1.999	−0.044	0.150	−0.068	0.326	−0.047	− 0.051
SDMT	−0.281	2.907	−0.105	0.899	−8.171*	23.727*	−0.802*	4.809*	−0.323	2.387	−1.086*	4.840
DSF	−0.039	0.425	0.017	−0.130	−0.698	2.184*	−0.065	0.454	−0.026	0.213	−0.069	0.338
LMT	−0.006	−0.113	0.075	−0.658	−1.188	4.261	−0.108	0.327	0.014	−0.394	−0.460	1.599

Abbreviations: MoCA: Montreal Cognitive Assessment; AVLT-IR: auditory verbal learning test-immediate recall; AVLT-SR: auditory verbal learning test-short recall; AVLT-LR: auditory verbal learning test-long recall; SDMT: symbol digit modalities test; DSF: digit span forwards; LMT: logical memory test; TC: total cholesterol; HDL-C: high-density lipoprotein cholesterol; LDL-C: low-density lipoprotein cholesterol; TG: triglycerides; Non-HDL-C: non- high-density lipoprotein cholesterol

β: unstandardized regression coefficients were obtained from mixed-effects regression models adjusted for sex, age, BMI, smoking and drinking status, diabetes, hypertension and coronary artery disease history and per se use of lipid-lowering medication, number of APOE ε4 alleles and intakes of energy, protein, carbohydrates, fat, SFA, PUFA, and MUFA

Elementary school: ≤6 years of education; Junior middle school: 7–9 years of education; Senior middle school: 10–12 years of education; College and above: at least college or university; ≥13 years of education)

∗*P* < 0.05

To summarize the effects most clearly, plots (Fig. 4 and 5) were generated using the predicted cognitive test scores associated with dietary cholesterol, egg intake and serum concentrations of cholesterol. Each graph depicted the significant quadratic or linear, longitudinal relationship between cognitive performance and cholesterol levels. In general, the plots showed that both lower and higher cholesterol/egg intakes were associated with poorer cognitive performance of global cognition, processing speed and executive function; serum concentrations of HDL-C within reference range was associated with better processing speed and executive function. Additionally, short and long recall of verbal memory was performed best at high and low levels of HDL-C and LDL-C/HDL-C ratio than at midrange. Last but not least, higher ratio of LDL-C/HDL-C and LDL-C levels was adversely associated with immediate recall of verbal memory and attention decline.

## Discussion

In this prospective study of 2514 community-dwelling participants initially with normal cognitive performance in middle-aged and elderly, we showed that higher levels of multiple cholesterol measurements were associated with higher risk of accelerated global cognitive decline. Moreover, we identified nonlinear or linear associations of dietary and serum cholesterol with domain-specific cognitive decline. Distribution of APOE ε4 risk alleles in our Asian population did not modify their associations. Subanalysis by educational group further demonstrated education-specific associations between cholesterol and cognition. This is, to our knowledge, the first report of nonlinear relations of both dietary and serum concentrations of cholesterol to longitudinal changes in cognitive performance.

Since the 2015–2020 Dietary Guidelines for Americans issued 2 seemingly contradictory statements concerned with dietary cholesterol [24], the worldwide controversy of dietary cholesterol has intensified primarily due to sparse data from human studies as well as contradictory conclusions resulting from between-study heterogeneity. It may not be applicable to follow the American dietary guidelines without regard to native conditions. Under the circumstances, the EMCOA study was conducted to investigate the impacts of dietary and serum cholesterol in middle-aged and elderly Chinese.

There are only two longitudinal human studies concerning the impact of dietary cholesterol on cognitive dysfunction but neither the risk of incident AD or dementia in Kuopio Ischaemic Heart Disease Risk Factor Study (KIHD) [2] nor cognitive decline in the Chicago Health and Aging Project (CHAP) [25] was associated with dietary cholesterol intake. In line with the results from aforementioned cohort studies, our longitudinal findings also report a non-significant association between dietary cholesterol/egg consumption and accelerated global cognitive decline with or without APOE adjustment. However, our previous studies have shown a beneficial association of dietary cholesterol with mild cognitive impairment (MCI) in cross-sectional settings [12, 26]. On the contrary, another two cross-sectional population-based study including participants with parallel age of ours from Netherlands [27] and Ireland [28] demonstrated higher dietary cholesterol intake was significantly associated with impaired cognitive performance.

As indicated by Smith and Refsum [29], the associations between the nutrient status and outcome usually follows a sigmoidal curve, which illustrates that additional nutrient intake is beneficial at low status but could be harmful at high intake; and it will have no effect at the plateau. Therefore, the ostensibly conflicting results across prior studies may not be truly contradictory. Higher consumption may correspond to the descending part of the curve while lower consumption may fell on the ascending part of the curve. Taking into consideration that dietary cholesterol intake increased dramatically in both Americans [30] and Chinese [31] and eggs were a major source of dietary cholesterol, a more cautious approach to dietary cholesterol and egg intake should be considered even though American and Chinese Dietary Guidelines dropped the recommendation on the cholesterol intake limit [32].

In regard to serum concentrations of cholesterol and cognitive changes, a large amount of research has demonstrated conflicting results and thus a small number of studies, including our cross-sectional studies [10], began to investigate nonlinear associations. The current study thus aimed to augment the understanding of nonlinear longitudinal cholesterol–cognition associations and served as an extension of nonlinear examination of non-HDL-C and ratio of LDL-C/HDL-C.

In both our studies, significant associations of HDL-C higher than 1.70 mmol/L with increased accelerated global cognitive decline and nonlinear relations of HDL-C with multiple domain-specific cognitive decline were observed. In contrast, longitudinal and cross-sectional studies from Wendell et al [8, 9] reported nonsignificant quadratic associations between HDL-C and cognitive performance. Moreover, another two large cohort studies from America [33] and France [34] also found HDL-C was not associated with 20-year cognitive decline or risk of incident dementia or its subtypes. Nevertheless, higher HDL-C is reported to be associated with better cognitive function in the Maine-Syracuse Study [35] and lower dementia risk in the Japan Public Health Centre-based prospective (JPHC) Study [36]. Owing to these controversial studies, the trend has turned to subclasses of HDL [37]. Ohtani et al. [38] have found significantly increased small-sized HDL particle levels but not HDL-C levels in MCI group compared with control group, suggesting potential associations between HDL subclasses and development of MCI. It is therefore conceivable that studies that examine associations between HDL-C and cognitive change may inevitably produce conflicting results, which may be obscured by a highly heterogeneous particle size of HDL. Further research is needed to clarify the association between lipoprotein particle characteristics of HDL, such as particle diameter and concentration, and cognitive changes.

In contrast to HDL-C, elevated TC and LDL-C at baseline was associated with greater cognitive decline regardless of being evaluated continuously or categorically, which were partially in support of specific adverse linear associations between LDL-C and attention decline. The Cox model findings for TC and LDL-C are consistent with the prospective study by Ma et al. [39]. It has been recognized that higher TC and LDL-C was cognitively detrimental due to correlated CVD risk among middle-aged and elderly individuals. However, a recent cross-sectional study has reported higher level of LDL-C may be considered as a potential protective factor against cognition decline [40]. Such evidence needs future replication but may have important clinical implications when taking that lower TC and LDL-C may be correspondingly detrimental owing to poor nutritional status and harmful effects on brain among the elderly.

The longitudinal associations of non-HDL-C, ratio of LDL-C/HDL-C with cognitive decline in cognitively healthy participants have been rarely examined and is still poorly understood, though both of them have been regarded as good predictors for CVD risk [41, 42]. As the sum of all the atherogenic lipoprotein particles other than the HDLs [20], higher serum levels of non-HDL-C was reported to be independent risk factors of cognitive impairment in patients with acute ischemic stroke [43] and MCI in patients with type 2 diabetes [44]. LDL-C/HDL-C ratio help to provide an estimate of how much cholesterol is removed by HDL and delivered to plaques via LDL. We demonstrated in Cox models that both of non-HDL-C and ratio of LDL-C/HDL-C could act as readily available methods for estimating risk of accelerated global cognitive decline in middle-aged and elderly Chinese. Besides, we also revealed quadratic and linear effects of LDL-C/HDL-C ratio with verbal memory, suggesting disordered cholesterol transport among atherogenic lipoprotein particles may be particularly detrimental to verbal memory. Measurement of non-HDL-C and ratio of LDL-C/HDL-C can be calculated from a usual lipid panel and consequently is simple and inexpensive. Where possible, their evaluation is needed as targets for intervention to reduce the risk of cognitive impairment.

Genetic variability of APOE is dependent on three alleles: ε2, ε3 and ε4, which combine to form six genotypes. It has been established that carriers of APOE ε4 have a greater risk of developing AD while APOE ε2 is considered protective [45]. However, the extent to which APOE genotype modulates associations of dietary cholesterol/egg intakes and serum cholesterol levels with cognitive decline remains relatively unknown. Our step-wise investigation of potential role of confounder and modifier examined how the cholesterol-cognition associations may vary by APOE ε4. Similar to studies in 1259 middle-aged and older men from Eastern Finland [2], the identified associations of cholesterol and cognitive decline were conserved after further adjusting for APOE ε4 among a middle-aged and elderly Chinese, possibly due to prominent distribution of APOE ε3.

The quantity of dietary cholesterol intake and serum lipid profiles may be affected by socio-economic factors [46, 47]. Our analysis provided such evidence that cholesterol/egg intakes and some serum cholesterol were not uniformly distributed across educational groups. With respect to consumption patterns, subjects with more years of education had more dietary cholesterol and egg intakes, which was different from older Australians with comparable age (55–65) reported by Thorpe et al [48]. They found those with higher consumption of red and processed meat had a lower level of education. When it comes to serum cholesterol, the population with higher levels of education presented a higher prevalence of altered cholesterol, inconsistent with Brazilian adult population aged 45 years old and older [49]. It may be attributed to the fact that participants aged 50–70 with lower education levels in our country, generally considered as lower socioeconomic status, were more likely to do physically intensive jobs while those with higher education levels were less likely to do physically active jobs and may be adapting unhealthy lifestyles. The subanalysis thus showed differential associations between cholesterol and cognitive function for different educational groups, which would be helpful to promote specific dietary instruction and lipid management according to educational background.

Strengths of this study include its longitudinal design, analysis of nonlinear effects of both dietary and serum cholesterol with consideration of multiple fractions, use of extensive cognitive tests and including data on the APOE genotype as well as per se use of a lipid-lowering medication for participants and examination measured in midlife, despite the fact that the median age of this cohort was much younger than the usual age of onset for MCI from nearly any cause including AD. Besides, the limitation of this investigation also includes its relatively shorter follow-up.

## Conclusion

Our findings highlight the complicated roles of dietary and serum cholesterol on cognitive decline in a particular population of middle-aged and elderly Chinese. Different cholesterol measurement appears to have varying degrees of associations for domain-specific achievement of better cognitive reserve. Therefore, interventions and policies regarding dietary instruction and lipid management must be tailored to address the specific challenges.

## Data Availability

The datasets during and/or analyzed during the current study available from the corresponding author on reasonable request.

## Abbreviations

AD: Alzheimer’s disease
APOE: Apolipoprotein E
AVLT: Auditory Verbal Learning Test
BMI: Body mass index
CHD: Coronary heart disease
DSF: Digit Span Forward
EMCOA: Effects and Mechanism investigation of Cholesterol and Oxysterol on Alzheimer’s disease
FFQ: Food frequency questionnaire
HDL-C: High-density lipoprotein cholesterol
LDL-C: Low-density lipoprotein cholesterol
LMT: Logical Memory Test
MoCA: Montreal Cognitive Assessment
non-HDL-C: Non-high-density lipoprotein cholesterol
SDMT: Symbol Digit Modalities Test
TC: Total cholesterol
TG: Triglycerides
WMS-RC: Wechsler Memory Scale—Revised, Chinese version
